# Long-Term Changes in Sarcopenia and Body Composition in Diabetes Patients with and without Charcot Osteoarthropathy

**DOI:** 10.1155/2022/3142307

**Published:** 2022-02-17

**Authors:** Michael Zaucha Sørensen, Rasmus Bo Jansen, Tomas Møller Christensen, Per E. Holstein, Ole Lander Svendsen

**Affiliations:** ^1^Department of Endocrinology, Bispebjerg Hospital, University of Copenhagen, DK-2400 Copenhagen NV, Denmark; ^2^Copenhagen Center for Wound Healing, Bispebjerg Hospital, University of Copenhagen, DK-2400 Copenhagen NV, Denmark

## Abstract

**Background:**

Charcot osteoarthropathy of the foot (COA) can currently only be treated using prolonged periods of immobilization of the affected extremity. Therefore, the hypothesis is that COA leads to altered body composition and increased sarcopenia.

**Objective:**

To investigate the changes over several years in sarcopenia, body composition, and fat distribution in diabetes patients with previous COA compared to diabetes patients without previous COA.

**Methods:**

Prospective observational clinical study. Twenty-one subjects were included and had two DXA scans done with mean 8.6-year intervals to compare changes in lean mass and fat distribution. The lean mass of limbs was used as an estimate of appendicular lean mass (aLM). Fat mass and aLM were then used to detect sarcopenic individuals using different methods. *Results and Conclusions*. As compared to baseline, both groups had significant loss of lean mass, and diabetics without COA had significant gain of total fat percentage. No statistically different prevalence of sarcopenia between the groups could be established. Likewise, no difference was found in total lean and fat mass changes. None of the groups had statistically significant changes of android fat distribution. As compared with published data on sarcopenia, people with diabetes might be more prone to sarcopenia than healthy individuals.

## 1. Introduction

Charcot osteoarthropathy of the foot (COA) is a rare but severe condition occurring in individuals with peripheral neuropathy, predominately patients with diabetes mellitus (DM). The mechanism behind the condition has not been clearly established, but microfractures, inflammation, and increased blood flow mediate collapse of the affected bones and destruction of surrounding joints in the foot [1]. DM patients with COA may have increased mortality and are at long-term risk of complications in the form of ulcers and/or amputations [2–4]. Thus far, we lack effective medical treatment for the condition and the treatment is weight off-loading of the foot using a cast, until the Charcot foot has healed, which may take as long as a year where the patient is immobilized to a degree [5, 6]. It is known that physical activity has a positive effect on muscle atrophy, i.e., sarcopenia [7, 8], and that adverse immobility can result in muscle atrophy [9]. The combination of less physical activity, as well as increased comorbidity, in DM patients with COA may have both short-term and long-term negative impacts on body composition, such as sarcopenia, increased body fat, and a more android fat distribution, which are known to increase the risk of cardiometabolic complications [10]. This was examined in a previous study showing no difference between the groups when using dual-energy X-ray absorptiometry (DXA) scans and derived sarcopenia rates [11].

The aim of this study was to compare changes over 8.6 years in body composition of diabetes patients with or without previous COA, the hypothesis being that DM patients with previous COA develop sarcopenia at a higher rate and become more obese with a higher abdominal/android fat distribution than DM patients without previous COA.

## 2. Materials and Methods

### 2.1. Subjects

The study population was based on the previously described population [11–13]. Subjects in all groups were invited to attend a second DXA scan 8 to 9 years after the first. Those who accepted and were scanned were then screened for possible excluding criteria in the form of amputation of one or more extremities or technical difficulties with the scan itself. This was based on the scan descriptions. The subjects were divided into two groups: DM patients with previous COA (DM+COA) and DM patients without previous COA (DM-COA). Both groups consisted predominately of males, so both genders were pooled in each group.

### 2.2. Measurements

Subjects were scanned using the same Lunar Prodigy GE DXA-scanner (Madison, WI, USA, using Encore 2005 software version 9.15.010). Whole-body scans were performed, and the scanner software then assessed the regional compositions of lean mass and fat mass as well as android and gynoid fat. Android fat is defined as the area around the waist between the midpoint of the lumbar spine and the top of the pelvis, while gynoid fat lies between the head of the femur and midthigh. For analyses, android and gynoid fats were expressed relative to total fat mass (as android or gynoid fat mass) divided by total fat mass. Finally, a truncal fat index was calculated as truncal fat mass divided by total fat mass. The trunk was defined by excluding the tissue of the extremities and the head. By DXA, lean tissue mass includes not only muscles but also water, skin, and connective tissue. The lean tissue mass of the limbs by DXA, called appendicular lean mass (aLM), can, however, be used as an estimate of the muscle mass in the limbs, and this was used for all sarcopenia calculations [14].

Ahead of the actual scans, subjects had their height and weight measured using the same hospital-grade electronic weight scales and wall-fixed height scales. Subjects were wearing light clothing and no shoes during these measurements.

### 2.3. Defining Sarcopenia

Sarcopenia was defined using the same two methods as previously described [11]. The first of these defines sarcopenia as aLM relative to height squared (aLM/h^2^), with values below 2 SD of the means of the population being the cut-off point for sarcopenia [15]. A previous study using a population of almost 3000 individuals aged 70-79 years established these cut-off points as 7.23 kg/h^2^ for men and 5.67 kg/h^2^ for women [16], and these were previously used [11]. This fitted the recommendations from the International Working Group on Sarcopenia [17]. However, more recent cut-off points of 7.00 kg/h^2^ for men and 5.50 kg/h^2^ for women were established using a population of 2371 individuals aged 20-93 years [18], and since these cut-off points are recommended in the updated guidelines from the European Working Group on Sarcopenia in Older People (EWGSOP) [19], these were chosen for this study.

A second method uses a linear regression to factor in fat mass in the definition of sarcopenia. As such, it uses the relationship between a predicted aLM on height and fat mass. Afterwards, a residual is calculated by subtracting the predicted aLM from the aLM observed on the DXA scans. Sarcopenia is then defined as values being below the lower 20^th^ percentile limit for the entire population included [16]. This method is quite rarely used and has not been widely implemented. However, a major benefit is the higher sensitivity in detecting sarcopenia among obese and overweight individuals as well as females. Considering the population included in this study, this method was included as well. Linear regressions were calculated for females and males separately at both baseline and follow-up. The previously found cut-off points defined above (-2.29 for males and -1.73 for females) were used to determine sarcopenia using this method.

### 2.4. Statistics

Statistical analyses were done using “R: A language and environment for statistical Computing version 4.0.3, R Foundation for Statistical Computing, Vienna, Austria.” General data handling as well as table generation was done using “Microsoft Excel Version 2010, build 13328.20356, by the Microsoft Corporation.” Figures were generated using “GraphPad Prism version 8.0.0 for Windows, GraphPad Software, San Diego, California USA.”

Data that was found to be normally distributed was compared using independent sample *t*-tests or paired *t*-tests where appropriate, while data that were not normally distributed were compared using Mann–Whitney *U* tests. To determine if data was normally distributed, the Shapiro–Wilks test for normality was used.

## 3. Results

### 3.1. Baseline Data of Study Populations

Of the 49 subjects in the original population [20], a total of 22 attended both scans. A table containing all raw data from the patient DXA scans is included as a supplementary file. A single patient was excluded due to technical difficulties with the second DXA scan resulting in abnormally low estimated total mass of the left upper extremity, leaving 21 subjects for the final analyses.  Table 1 shows a summary of the demographic information of the two groups at the baseline and the changes at follow-up scans. The follow-up time between the two scans was statistically significantly different for the two groups, with the DM+COA group having a mean one-year longer follow-up. The populations in general were mostly overweight, with 81 percent of the study population having a BMI of >25, while 33 percent had a BMI of >30.

Type 1 diabetes was more prevalent in the DM+COA group (45.5% vs 20%). Out of the total population, 33.3% of the subjects had type 1 diabetes.

For the DM+COA group, the time from initial diagnosis until performing the first DXA scan varied from 0.5 to 13 months, with a mean duration of 3.66 ± 4.36 months.

Total immobilization for COA was between 5 and 14.5 months with a mean of 9.36 ± 5.18 months.

### 3.2. Comparison of Anthropomorphic Data

**Figure 1:**
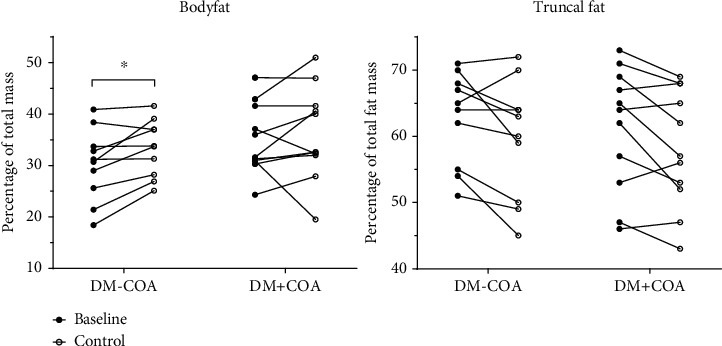
Changes in fat distribution: individual changes in body fat (defined as percentage of total soft tissue mass) and truncal fat (defined as percentage of total fat mass) from baseline to follow-up after 8.6 years, in diabetes patients without (DM-COA) or with (DM+COA) a previous Charcot foot. ∗: significant increase from baseline (*p* = 0.01). Otherwise, there were no significant changes from baseline or changes between DM-COA and DM+COA (*p* > 0.05).

There was a significant loss of total lean mass for both groups (DM-COA: *p* = 0.0005; DM+COA: *p* = 0.02). Likewise, both groups had significant loss of aLM (DM-COA: *p* = 0.0001; DM+COA: *p* = 0.01) (Table 1). The DM-COA group had a significant gain of body fat percentage with *p* = 0.01 but this did not translate to significant gain of truncal fat (Figure 1). No other parameters had statistically significant changes over time. There were no statistically significant differences in changes between the DM+COA and DM-COA groups.

### 3.3. Sarcopenia Calculations

Sarcopenia was defined using both methods described. When using the aLM/h^2^ method of detecting sarcopenia, a decline of −0.63 kg/h^2^ ± 0.31, *p* = 0.0001, was seen in the DM+COA group and −0.88 kg/h^2^ ± 0.97, *p* = 0.02, in the DM-COA group. Comparing the two groups showed no significant difference in the decline in aLM/h^2^ (*p* = 0.45).

As for the residuals, linear regressions were as follows:

Males:

*Baseline*: −13.26 + 19.57∗(height in meters) + 0.18∗(fat mass in kilograms).

*Follow-up*: −33.2 + 30.12∗(height in meters) + 0.10∗(fat mass in kilograms).

Females:

*Baseline*: −24.63 + 18.36∗(height in meters) + 0.4∗(fat mass in kilograms).

*Follow-up*: −118.79 + 96.29∗(height in meters) − 0.59∗(fat mass in kilograms).

Neither group had any significant loss nor gain in residual values (DM+COA: *p* = 0.40; DM-COA: *p* = 0.85). Likewise, no significant difference in the change of residual values was found when comparing the two groups (*p* = 0.85).

**Figure 2:**
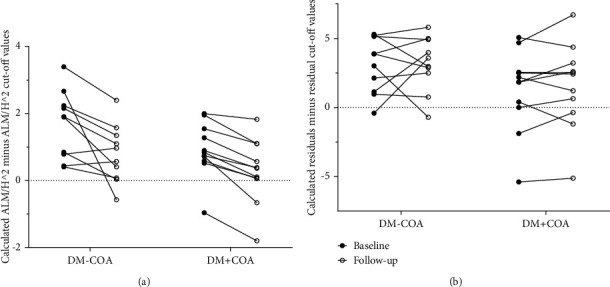
Changes in muscle mass using different methods: Individual changes in measures of appendicular muscle mass and sarcopenia from baseline to follow-up after 8.6 years in diabetes patients without (DM-COA) or with (DM+COA) a previous Charcot foot. (a) Shows aLM/h2 results expressed as individual aLM/h2 values minus reference cut-off values for sarcopenia, and (b) shows residual results using the same method. A value less than 0 is therefore below cut-off and indicates sarcopenia. There were no significant changes from baseline to follow-up within or between the groups (*p* > 0.05).

Differences from cut-off values in absolute numbers for both methods are shown in Figure 2.

For the DM+COA group, there was no significant correlation between time immobilized and change in aLM/h^2^ and residual values.

**Figure 3:**
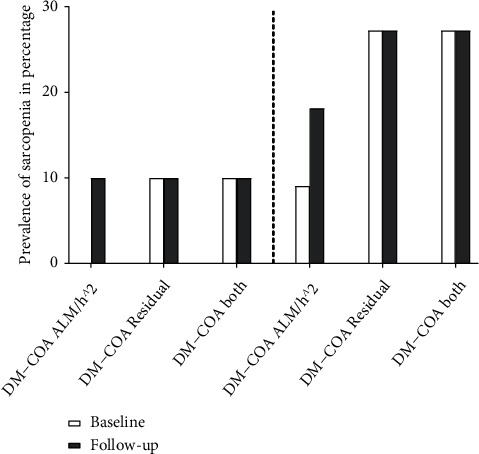
Sarcopenia prevalence using different methods: prevalence of sarcopenia shown using different methods at baseline and at follow-up after 8.6 years in diabetes patients without (DM-COA) or with (DM+COA) a previous Charcot foot. “Both” methods indicate sarcopenia detected using any of the aLM/h2 of residual methods. There were no significant differences in the prevalence of sarcopenia between the groups (*p* > 0.05).

A prevalence of sarcopenia of 27 percent was found in the DM+COA group at follow-up, with sarcopenic individuals being defined as subjects being below cut-off using either of the two models, with the control group having a prevalence of 10 percent. This was unchanged for the both groups (Figure 3). Using the residual method, the prevalence of sarcopenia remained static at 27 percent for the DM+COA group and 10 percent for the DM-COA group. Meanwhile, there was an increase in the prevalence of sarcopenia when using the aLM/h^2^ method from 9 percent in the DM+COA group and 0 percent in the DM-COA group to 18 percent and 10 percent, respectively. Analysing the prevalence of sarcopenia using any of the methods at either timepoint using Fisher's exact test showed no statistically significant differences.

There was no significant difference in the prevalence of sarcopenia when comparing type 1 and type 2 diabetics both at baseline and follow-up using any of the above methods.

## 4. Discussion

To our knowledge, this is the first study to examine prospective changes to muscle mass, body composition, and fat distribution in DM patients with COA. Since COA is treated using prolonged immobilization which could cause atrophy, our hypothesis was that diabetes patients with COA would be more prone to developing sarcopenia, both short term due to the immobilization of a lower extremity and also long term due to negative changes to lifestyle associated with the condition.

However, our results do not support this theory. While more individuals met the criteria for sarcopenia in the COA group using the different methods, this did not translate to statistically significant different prevalence. It is possible the relatively low number of subjects included in the study is the cause of this. Prior to performing analyses, no sample size calculation was done; instead, as many subjects as possible were included from the original population as previously described. This is naturally a big limitation to the design and results of this study.

Pooling the subjects as a group with DM in common and examining the number of sarcopenic individuals reveal a prevalence of sarcopenia of 19 percent. This is a markedly higher prevalence compared to a population of healthy, older community-dwelling Canadians [21], as well as a population of subjects with osteoporosis [22], although no statistical difference can be established when comparing the data. Meanwhile, two-thirds of all the subjects in this study had a lower calculated aLM/h^2^ value compared to the age and gender adjusted means that was found in a population of healthy individuals [23]..

None of the groups had increased android fat deposition. It was expected that especially the DM+COA subjects would gain fat mass as previously described, which would translate to gain of android fat mass. On the contrary, the DM-COA patients had a gain of fat mass but still did not have increased amount of android fat. Due to the relation between android fat mass and cardiometabolic risk factors [10], these results, although not showing a strict positive tendency, at least show that COA does not seem to negatively influence this and that status quo for this parameter is maintained.

As for the models used to determine sarcopenia, there was a marked difference in how many subjects were defined as sarcopenic when comparing the aLM/h^2^ method with the residual method. This was especially apparent with the COA group at both baseline and follow-up. The aLM/h^2^ model is what is predominantly used when using DXA scans to determine sarcopenia in most literature. With that said, the advantages of defining sarcopenia as relative to fat mass still seem logical and it was demonstrated that changes in BMI had a drastic effect on the prevalence of sarcopenia using different methods, with the residuals method being able to detect sarcopenia at a much higher rate in overweight and obese individuals [16], which is what is predominately the case in this study group. The most pragmatic approach would perhaps be to do both tests when determining whether an individual is sarcopenic or not, although this would potentially result in an abnormally high number of positives. This would also require an unequivocal cut-off value for the residual model to be used as gold standard.

## 5. Conclusions

Over 8.6 years of follow-up, diabetes patients with a previous Charcot foot had significant loss of estimated appendicular muscle mass, but no significant changes in body fatness nor fat distribution, and these changes were similar to those of diabetes patients without a previous Charcot foot. The diabetes patients, with or without a previous Charcot foot, might be more prone to sarcopenia compared to healthy individuals.

## Figures and Tables

**Table 1:** Subject demographics: baseline values and changes at follow-up after 8.6 years in diabetes patients without (DM-COA) or with (DM+COA) a previous Charcot foot. Data expressed as means ± SD. Truncal, android, and gynoid body fat percentages are all calculated relative to total fat mass while body fat percentage is calculated relative to total soft tissue mass. aLM is appendicular lean mass. *p* values compare the changes at follow-up between the two groups. ∗: significant changes from baseline (*p* < 0.05).

	Baseline		Changes at follow-up		
Parameter	DM-COA	DM+COA	DM-COA	DM+COA	*p* value
Age (years)	62.5 ± 4.2	58.8 ± 8.4	8.1 ± 0.7	9.1 ± 0.3	0.001
Diabetes type 1/2	2/8	5/6	2/8	5/6	*—*
Female/male	1/9	3/8	1/9	3/8	*—*
Height (m)	1.8 ± 0.1	1.8 ± 0.1	0 ± 0.0	0 ± 0.0	0.51
Weight (kg)	91.9 ± 15.9	90.0 ± 13.1	0.2 ± 6.5	−1.8 ± 6.8	0.50
BMI	29.5 ± 5.3	27.8 ± 1.9	0.2 ± 2.2	−0.4 ± 2.0	0.54
Fat mass (kg)	27.6 ± 10.3	29.8 ± 4.6	2.5 ± 4.7	0.7 ± 7.0	0.49
Body fat (%)	30.2 ± 7.0	34.9 ± 6.7	3.2 ± 3.3^∗^	1.2 ± 5.7	0.34
Truncal fat (%)	62.7 ± 7.1	61.3 ± 9.3	−3.1 ± 4.7	−3.1 ± 4.2	0.99
Android fat (%)	11.6 ± 1.7	11.0 ± 2.1	-0.8 ± 0.8	-0.2 ± 1.2	0.16
Gynoid fat (%)	15.6 ± 3.3	15.4 ± 2.8	-1.8 ± 1.4	-0.8 ± 1.4	0.15
Android/gynoid ratio	0.8 ± 0.3	0.8 ± 0.2	0.0 ± 0.1	0.0 ± 0.1	0.63
Lean mass (kg)	61.2 ± 6.7	57.0 ± 12.2	−2.4 ± 2.7^∗^	−2.4 ± 1.6^∗^	0.98
aLM (kg)	26.8 ± 3.4	24.8 ± 6.1	−2.8 ± 2.7^∗^	−2.2 ± 1.2^∗^	0.48

## Data Availability

All data is drawn from DXA scan reports containing patient sensitive information; as such, it is not publicly available. A table containing all raw data used is submitted as a supplementary file along with the manuscript.

## Supplementary Materials

Supplementary Materials A table containing all raw data used for analyses has been submitted along with the primary manuscript. This includes anthropomorphic data, duration of immobilization, time to DXA scan, and calculated aLM/h^2^ and residual values.
