# Supplementary material for: Long-Term Changes in Sarcopenia and Body Composition in Diabetes Patients with and without Charcot Osteoarthropathy
Source: J Diabetes Res. 2022 Feb 17;2022:3142307. doi: 10.1155/2022/3142307 (PMC8872651; doi:10.1155/2022/3142307)
Supplement: Supplementary Materials — A table containing all raw data used for analyses has been submitted along with the primary manuscript. This includes anthropomorphic data, duration of immobilization, time to DXA scan, and calculated aLM/h2 and residual values. [file 3142307.f1.pdf]

Baseline data:

Subject ID	Group	Age	DM Type	Time from diagnosis to DXA (months)	Total duration of immobilisation (months)	Gender	Height	Weight	BMI	Arms	Legs	Android	Gynoid	A/G	Lean Mass	% Fat	Fat mass	aLM	Pred. aLM	aLM/H^2	Residual
1	CharcotB	47.7	2	11	13	M	1.93	102	27.38328546	7.714	21.703	40	32.8	1.219512195	66.679	30.9	29.787	29.417	29.18274763	7.897393219	0.23425237
2	CharcotB	62.4	2	0.5	7	F	1.65	82	30.11937557	4.443	12.55	48.3	51	0.947058824	41.957	47.1	37.333	16.993	16.5515118	6.241689624	0.4414882
6	CharcotB	62.5	2	0.5	6	M	1.9	100	27.70083102	8.028	24.331	44.2	27.5	1.607272727	68.87	31.3	31.359	32.359	28.74893791	8.963711911	3.61006209
7	CharcotB	76.2	1	3	3	F	1.5	63	28	2.494	11.046	43.2	48.4	0.892561983	34.552	41.6	24.633	13.54	13.3301868	6.017777778	0.2098132
8	CharcotB	53	2	1.5	5	M	1.82	93	28.07631929	7.637	18.341	44.9	32.8	1.368902439	58.298	36	32.781	25.978	27.34699469	7.842651854	-1.36899469
9	CharcotB	59.3	1	1.25	11	M	1.76	85	27.44059917	6.477	17.443	43.8	30.3	1.445544554	55.342	31.6	25.626	23.92	25.56801674	7.722107438	-1.64801674
10	CharcotB	55.7	1	5	14	M	1.88	88	24.89814396	6.576	14.765	45.3	41	1.104878049	51.755	37.1	30.483	21.341	28.28947667	6.038082843	-6.94847667
12	CharcotB	49.8	1	2.5	6	F	1.71	82	28.04281659	5.1	15.526	44.8	51.7	0.866537718	45.828	42.9	34.39	20.626	21.614061	7.05379433	-0.988061
13	CharcotB	58.6	1	0.5	4.5	M	1.92	92	24.95659722	7.406	20.589	30	29.6	1.013513514	65.493	24.3	21.05	27.995	28.2186385	7.594129774	-0.2236385
15	CharcotB	68.3	2	1.5	14.5	M	1.86	107.9	31.18857671	7.564	23.588	44.1	23	1.917391304	71.502	30.3	31.094	31.152	27.96160006	9.004509192	3.19039994
18	CharcotB	53.1	2	13	19	M	1.87	99.5	28.45377334	6.787	22.162	40.1	31.3	1.28115016	66.2	31	29.71	28.949	28.0301019	8.278475221	0.9188981
35	ControlB	59.8	2			F	1.92	114	30.92447917	8.38	25.396	47	33.3	1.411411411	72.059	33.7	36.617	33.776	33.4400472	9.162326389	0.9188981
38	ControlB	62.9	2			M	1.7	107	37.02422145	6.721	18.989	44	44.1	0.997732426	61.401	40.9	42.413	25.71	25.90766637	8.896193772	0.3359528
39	ControlB	61.3	2			M	1.77	85	27.13141179	6.775	17.809	42.3	33.3	1.27027027	56.66	30.7	25.111	24.584	25.71341639	7.847042676	-0.19766637
40	ControlB	54.5	1			M	1.68	70	24.8015873	6.856	18.294	29	27.6	1.050724638	52.963	21.4	14.397	25.15	23.04658653	8.910856009	-1.12941639
41	ControlB	67.5	2			M	1.8	89	27.4691358	7.334	17.911	40.4	26	1.553846154	60.382	29	24.649	25.245	26.24545001	7.791666667	2.10341347
42	ControlB	65.8	2			M	1.79	109	34.01891327	7.213	22.38	43.4	34.3	1.265306122	70.706	32.8	34.48	29.593	26.9244232	9.235978902	-1.00045001
43	ControlB	65.5	2			M	1.72	79	26.70362358	6.057	15.961	35.7	34.2	1.043859649	52.831	31.2	24.008	22.018	24.66095192	7.442536506	2.6685768
44	ControlB	57.8	2			M	1.7	109	37.71626298	6.706	21.254	50.1	38.9	1.287917738	64.365	38.4	40.104	27.96	25.70334296	9.674740484	-2.64295192
45	ControlB	62.6	2			M	1.81	89	27.16644791	7.777	21.374	38.8	23	1.686956522	63.868	25.6	22.019	29.151	26.20369331	8.898080034	2.25665704
48	ControlB	67	1			M	1.81	71	21.67211013	6.621	17.67	24.8	25.8	0.96124031	56.253	18.4	12.643	24.291	25.37401107	7.414608834	2.94730669

Follow-up data:

Subject ID	Gruppe	Age	DM Type	Time from diagnosis to DXA (months)	Total duration of immobilisation (months)	Gender	Height	Weight	BMI	Arms	Legs	Android%	Gynoid%	A/G	Lean Mass	% Fat	Fat mass	aLM	Pred aLM	aLM/H^2	Residual
1	CharcotF	56.4	2	11	13	M	1.93	102	27.38328546	7.49	19.947	24.9	21.9	1.136986301	65.027	19.5	15.702	27.437	35.1316	7.365835324	-7.6946
2	CharcotF	71.6	2	0.5	7	F	1.65	82	30.11937557	3.884	12.162	48.9	49.2	0.993902439	41.891	47	37.216	16.046	18.294413	5.893847567	-2.248413
6	CharcotF	71.9	2	0.5	6	M	1.88	100	28.29334541	6.938	21.693	39.9	29	1.375862069	66.292	32	31.172	28.631	33.4256	8.100667723	-4.7946
7	CharcotF	84.9	1	3	3	F	1.5	57	25.33333333	3.405	9.155	38.8	46.7	0.830835118	32.758	41.6	23.363	12.56	11.9656765	5.582222222	0.5943235
8	CharcotF	61.8	2	1.5	5	M	1.82	92	27.77442338	7.168	16.384	51.5	34.5	1.492753623	53.779	40	35.84	23.552	30.8184	7.110252385	-7.2664
9	CharcotF	68.9	1	1.25	11	M	1.76	85	27.44059917	5.973	13.754	48.3	35.4	1.36440678	49.713	40.6	33.982	19.727	28.3112	6.368478822	-8.5842
10	CharcotF	64.7	1	5	14	M	1.88	88	24.89814396	5.572	12.797	32.6	40.9	0.797066015	49.293	32.3	23.501	18.369	32.2256	5.197204617	-13.8566
12	CharcotF	58.9	1	2.5	6	F	1.71	90	30.77870114	4.613	14.708	55.6	54.6	1.018315018	44.629	51	46.461	19.321	18.6610365	6.607503163	0.6599635
13	CharcotF	67.8	1	0.5	4.5	M	1.92	92	24.95659722	5.497	20.545	30.4	31.2	0.974358974	63.965	27.9	24.805	26.042	33.8304	7.064344618	-7.7884
15	CharcotF	77.6	2	1.5	14.5	M	1.86	107.9	31.18857671	7.79	22.775	44.4	26.8	1.656716418	68.561	32.6	33.113	30.565	33.6132	8.834836397	-3.0482
18	CharcotF	62.3	2	13	19	M	1.87	99.5	28.45377334	5.761	20.72	40.8	31.9	1.278996865	64.298	32.6	31.057	26.481	33.0744	7.572707255	-6.5934
35	ControlF	68.3	2			M	1.92	114	30.92447917	8.346	22.434	46.9	32.6	1.438650307	68.908	33.8	35.118	30.78	36.0304	8.349609375	-5.2504
38	ControlF	70.4	2			F	1.7	107	37.02422145	6.205	16.633	43	44.1	0.975056689	55.289	41.6	39.364	22.838	21.847512	7.902422145	0.990488
39	ControlF	69.8	2			M	1.77	90	28.72737719	6.697	15.372	46.8	37.9	1.234828496	54.245	39.1	34.876	22.069	29.1124	7.04427208	-7.0434
40	ControlF	62.8	1			M	1.68	70	24.8015873	6.271	16.6	31.9	29.4	1.085034014	52.677	26.9	19.414	22.871	24.4016	8.103387188	-1.5306
41	ControlF	75.8	2			M	1.78	93	29.3523545	7.025	18.227	45.3	28.5	1.589473684	61.696	33.7	31.349	25.252	29.7136	7.969953289	-4.4616
42	ControlF	73.1	2			M	1.79	109	34.01891327	6.069	21.418	43.4	34.5	1.257971014	69.11	37	40.662	27.487	31.6148	8.578696046	-4.1278
43	ControlF	73.9	2			M	1.72	79	26.70362358	6.512	15.883	34.3	34.6	0.99132948	54.569	31.3	24.853	22.395	26.5064	7.569970254	-4.1114
44	ControlF	67.1	2			M	1.7	105	36.33217993	4.567	14.027	42.2	32	1.31875	60.898	37	35.795	18.594	28.504	6.433910035	-9.91
45	ControlF	70.1	2			M	1.81	89	27.16644791	6.618	17.65	38.4	22	1.745454545	57.731	28.2	22.628	24.268	30.2172	7.407588291	-5.9492
48	ControlF	74.4	1			M	1.79	70	21.84700852	5.748	16.943	27.3	29.1	0.93814433	52.75	25.1	17.635	22.691	27.7148	7.081863862	-5.0238
